# Postoperative Radiochemotherapy Using Modern Radiotherapy Techniques in Elderly Patients with Head and Neck Squamous Cell Carcinoma: The Challenge of Weighing Up Benefits and Harms of Treatment Modalities in Clinical Practice

**DOI:** 10.3390/cancers13143384

**Published:** 2021-07-06

**Authors:** Martin Leu, Christoph Patzer, Manuel Guhlich, Jacqueline Possiel, Yiannis Pilavakis, Markus Anton Schirmer, Stefan Rieken, Leif Hendrik Dröge

**Affiliations:** 1 Department of Radiotherapy and Radiation Oncology, University Medical Center Göttingen, Robert-Koch-Str. 40, 37075 Göttingen, Germany; martin.leu@med.uni-goettingen.de (M.L.); christoph.patzer@stud.uni-goettingen.de (C.P.); manuel.guhlich@med.uni-goettingen.de (M.G.); jacqueline.possiel@med.uni-goettingen.de (J.P.); mschirmer@med.uni-goettingen.de (M.A.S.); stefan.rieken@med.uni-goettingen.de (S.R.); 2 Department of Otorhinolaryngology, University Medical Center Göttingen, 37075 Göttingen, Germany; yiannis.pilavakis@med.uni-goettingen.de

**Keywords:** head and neck, squamous cell cancer, postoperative treatment, radiochemotherapy, elderly patients, geriatric oncology, comorbidities, acute toxicity, late toxicity, survival

## Abstract

**Simple Summary:**

Locally advanced head and neck squamous cell carcinomas (HNSCC) are often managed with surgery and postoperative radiochemotherapy (RCT). Until now, a deeper understanding of specific management strategies for elderly patients was lacking. In the present study, we compared patients ≥70 years of age and younger patients treated with postoperative RCT for HNSCC. All patients were treated with modern radiotherapy techniques (IMRT/VMAT). Elderly patients had more comorbidities. In addition, they less frequently received concomitant systemic treatment. The rates of mucositis and dermatitis were lower in patients ≥70 years. Elderly patients had significantly worse overall survival and progression-free survival. Locoregional and distant control were comparable in elderly and younger patients. In conclusion, postoperative radiochemotherapy is a safe and effective treatment option in patients ≥70 years. In light of comorbidities and poor survival rates, potential benefits and harms of radiotherapy and concomitant systemic treatment should be weighed carefully for this age group.

**Abstract:**

Locally advanced head and neck squamous cell carcinomas (HNSCC) are often managed with surgery followed by postoperative radiochemotherapy (RCT). With the general increase in life expectancy, the proportion of elderly patients with HNSCC is expected to grow rapidly. Until now, a deeper understanding of specific management strategies for these patients in clinical routine was lacking. In the present study, we compared elderly patients (≥70 years, *n* = 52) and younger patients (*n* = 245) treated with postoperative RCT for HNSCC at our tertiary cancer center. All patients were irradiated with modern radiotherapy techniques (IMRT/VMAT). Patients ≥70 years of age had more comorbidities. Additionally, elderly patients less frequently received concomitant systemic treatment. The rates of mucositis and dermatitis were lower in patients ≥70 years. Elderly patients had significantly worse overall and progression-free survival. Locoregional and distant control were comparable in elderly and younger patients. In conclusion, postoperative RCT is a safe and effective treatment option in patients ≥70 years. In light of comorbidities and poor overall survival rates, benefits and harms of radiotherapy and concomitant systemic treatment should be weighed carefully. When exclusively applying up-to-date radiotherapy techniques with, at the same time, careful use of concomitant systemic therapy, favorable acute toxicity profiles are achieved.

## 1. Introduction

Head and neck squamous cell carcinomas (HNSCC) caused 450,000 deaths worldwide in 2018 [1]. The incidence is rising and an increase of 30% is predicted until 2030 [1]. Locally advanced HNSCC are often managed with intensive multimodal treatment, including surgery followed by postoperative radiochemotherapy (RCT) [2,3,4]. Nevertheless, long-term outcomes remain poor, with 10-year overall survival and disease-free survival rates of less than 30% [5]. At the same time, local treatment is associated with high rates of acute and long-term morbidity [6,7,8].

In elderly patients, HNSCC are rare [9]. Less than 20% of the HNSCC patients are >70 years of age [9]. At the same time, regarding the general increase of life expectancy, the proportion of elderly patients with HNSCC is expected to grow rapidly [1,10]. In the major clinical trials on treatment strategies in HNSCC, these patients were either excluded or were underrepresented [3,4,11]. This is the case for earlier trials which established the indications for postoperative RCT as well as for contemporary prospective trials on treatment strategies in HNSCC [3,4,11,12]. Recently, several mostly retrospective studies focused on outcomes in elderly patients and found evidence that specific management approaches are absolutely needed [13,14,15]. Nevertheless, until now, a deeper understanding of the implications of the findings for clinical routine was lacking [15].

In the present study, we analyzed patients treated with postoperative RCT for HNSCC at our tertiary cancer center. We compared baseline characteristics, treatment-associated toxicities, and outcomes between elderly patients ≥70 years of age and patients <70 years of age in a modern cohort. All patients were treated with either conventional intensity-modulated radiotherapy (IMRT) or volumetric modulated arc therapy (VMAT).

## 2. Patients and Methods

### 2.1. Patient Cohort

We retrospectively reviewed our clinic’s medical records for patients treated with postoperative RCT for non-metastatic HNSCC. Patients were included beginning in 05/2008, when new linear accelerators were installed and treatment with IMRT/VMAT was standardly used in our radiotherapy department. Before surgery, all patients underwent staging examinations (CT scan of the head and neck, endoscopic head and neck examination, chest X-ray and abdominal ultrasound or CT examination of thorax and abdomen). The oncological strategies were discussed (both pre- and post-surgery) in the local multidisciplinary tumor board consisting of experienced radiation oncologists, head and neck surgeons, neuroradiologists, and pathologists. The procedures were performed in accordance with the guidelines [16,17,18,19]. This retrospective study was conducted after authorization by the local ethics committee (University Göttingen Medical Center, number 6/1/21).

### 2.2. Radiotherapy

Postoperative radiotherapy was standardly performed in patients with ≥pT3, pN+, or R1 tumors [16,17,18,19]. In addition, postoperative radiotherapy was discussed on an individual basis if other risk factors were present (e.g., L1, V1 status, patients with single positive lymph nodes without extracapsular extension, patients with recurrent disease) [16,17,18,19]. Beschel et al. previously described the radiotherapy procedures [20]. Before radiotherapy, patients received a comprehensive dental evaluation by the University Medical Center dentists. Here, a dental splint was individually adapted. Patients underwent planning CT scans of the head and neck region with customized thermoplastic masks. The clinical target volume included the primary tumor region and the neck. The contours were generated based on the respective guidelines [21,22,23]. The planning target volume was generated using a 10 mm margin in all directions. All patients were treated either with conventional IMRT or with VMAT (RapidArc^®^, Varian Medical Systems, Palo Alto, CA, USA) with daily on-board imaging. Treatment plans were calculated using Eclipse (Varian Medical Systems, Palo Alto, CA, USA). The organs at risk constraints were used according to the QUANTEC recommendations [24,25,26].

### 2.3. Concomitant Systemic Treatment

The procedures in systemic treatment were already described previously [20,27,28]. Concomitant systematic treatment was standardly applied in patients with extracapsular extension and R1 tumors. Additionally, patients were treated with concomitant chemotherapy in case of further risk factors (e.g., ≥2 positive lymph nodes, UICC stages III–IV). The decision was left at the discretion of the individual treating physician [2,3,4]. The pre-treatment examinations were a complete blood cell count with clinical chemistry, an electrocardiogram, an audiometry test and a creatinine clearance from a 24 h urine collection. In patients with creatinine clearance of 60–<70 mL/min, concomitant cisplatin was used with strict indication. In cases with creatinine clearance of <60 mL/min, cisplatin was omitted. The cisplatin regimen (e.g., weekly or daily) was set by the treating physician on an individual basis. In cases with contraindications against cisplatin, cetuximab was applied according to the schedule by Bonner et al. [29].

### 2.4. Follow-Up Procedures and Toxicity Scoring

Patients who received concomitant chemotherapy were hospitalized for the RCT initiation. A minimum of weekly visits with assessment of toxicities were performed during RCT; including a complete blood cell count and an assessment of clinical chemistry. The toxicities were scored in accordance with the CTCAE criteria v5.0 (acute toxicities, [30]) and in accordance with the LENT/SOMA criteria (late toxicities, [31]). After RCT, follow-up with anamnesis and clinical examination was performed in 18-month intervals for a total of 5 years. Additionally, patients regularly presented to the treating head and neck surgeon.

### 2.5. Statistics

The software SPSS (v. 26) was used for data administration and statistical analysis. The survival curves were generated and compared with log-rank test using the software ‘R’ (v. 4.0.2, plugin ‘KMWin’ [32]). The chi-square test and the Mann–Whitney U test were applied when comparing baseline and RCT characteristics for patients ≥70 years vs. <70 years of age and for the comparison of patients ≥70 years of age who underwent radiotherapy alone vs. RCT. The survival times were counted from the day of histopathological tumor diagnosis. We evaluated overall survival (OS; event: patient death due to any cause), progression-free survival (PFS; events: patient death due to any cause and any tumor progression), time to recurrence (TTR; events: local, regional or distant tumor progression, death due to HNSCC), locoregional control (LRC; events: local or regional tumor progression), and distant control (DC; event: occurrence of distant metastases).

## 3. Results

### 3.1. Patient and Tumor Characteristics

In total, we included 297 consecutive patients. The cut-off for the definition of ‘elderly patients’ was set at the age of 70 years. This cut-off has been reported to be most frequently used in geriatric oncology and to have clinical relevance [33,34]. In the whole cohort, 245 patients (82.5%) were younger than 70 years and 52 patients (17.5%) were older than or equal to 70 years old. Patients were treated from May 2008 to November 2019. The median age was 60.0 years (range, 23.0–85.0 years). The median follow-up was 37.0 months (range, 3.0–147.0 months). When comparing the treatment groups, patients ≥70 years of age had more comorbidities and fewer tumors with nodal involvement. Please see Table 1 for further details.

### 3.2. Radiochemotherapy

All patients were treated with IMRT (*n* = 126, 42.4%) or VMAT (*n* = 171, 57.6%). Both the median planned and administered radiotherapy doses were 64.0 Gy (range, 54.0–66.0 Gy and 38.0–66.0 Gy). In detail, the planned radiotherapy doses in 182 patients were 64 Gy (*n* = 178) or 66 Gy (*n* = 4) with simultaneous irradiation of primary tumor region (2.0 Gy/fraction, 30 fractions), affected lymph node regions (1.92 Gy/fraction, 30 fractions), and elective nodes (1.8 Gy/fraction, 30 fractions), followed by a sequential boost of 2*2 Gy (*n* = 178) or 3*2 Gy (*n* = 4) to the primary tumor. The planned dose in 112 patients was 62.4 Gy with simultaneous irradiation of primary tumor region (2.08 Gy/fraction, 30 fractions), affected lymph node regions (1.92 Gy/fraction, 30 fractions), and elective nodes (1.8 Gy/fraction, 30 fractions). In one patient, the planned dose was 63.0 Gy (simultaneous irradiation of primary tumor region/affected lymph node regions up to 57.6 Gy in 30 fractions [1.92 Gy/fraction] and irradiation of elective nodes up to 54 Gy in 30 fractions [1.8 Gy/fraction], followed by a sequential boost with 3*1.8 Gy to the primary tumor region). One patient had a planned dose of 60.0 Gy (30 fractions of 2 Gy/fraction to the primary tumor region and 54 Gy [1.8 Gy/fraction] to the neck). In one patient, the planned dose to the primary tumor region and to the neck was 54 Gy in 1.8 Gy fractions. A total of 282 patients (94.9%) completed radiotherapy as prescribed. In the whole study cohort, a concomitant systemic treatment was given in 223 patients (75.1%). Elderly patients less frequently received a concomitant systemic treatment. Additionally, the percentage of patients ≥70 years receiving cetuximab instead of cisplatin was higher compared to younger patients. Please see Table 2 for further details.

### 3.3. Toxicities

In the whole study cohort, 167/297 patients (56.2%) experienced acute organ toxicities ≥grade 3. We observed ≥grade 3 mucositis in 32/297 patients (10.8%), ≥grade 3 dermatitis in 7/297 patients (2.4%), and ≥grade 3 dysphagia in 156/297 patients (52.5%). Hematologic toxicities of ≥grade 3 were documented in 73/297 patients (24.6%). Late toxicities ≥grade 3 were registered in 34/267 patients (12.7%, information on late toxicity missing in 30 patients). When comparing patients ≥70 years of age vs. <70 years of age, elderly patients experienced mucositis and dermatitis (grades 1–4) less frequently. There were no differences in the rates of high-grade (≥grade 3) mucositis and dermatitis between the age groups. Please see Table 3 for further details. Additionally, in patients ≥70 years of age, there were no differences in acute and late toxicities between patients who received radiotherapy alone and patients who received RCT (Supplementary Table S1).

### 3.4. Survival

**Figure 1:**
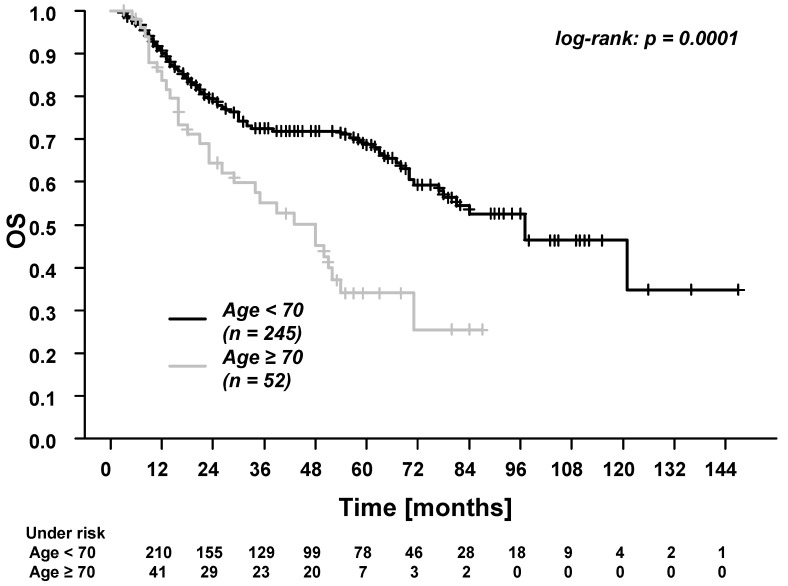
Overall survival (OS) for patients <70 years vs. patients ≥70 years of age. The 3-year and 5-year OS for patients <70 years were 72.5% and 68.7%. The 3-year and 5-year OS for patients ≥70 years were 55.2% and 33.8%.

**Figure 2:**
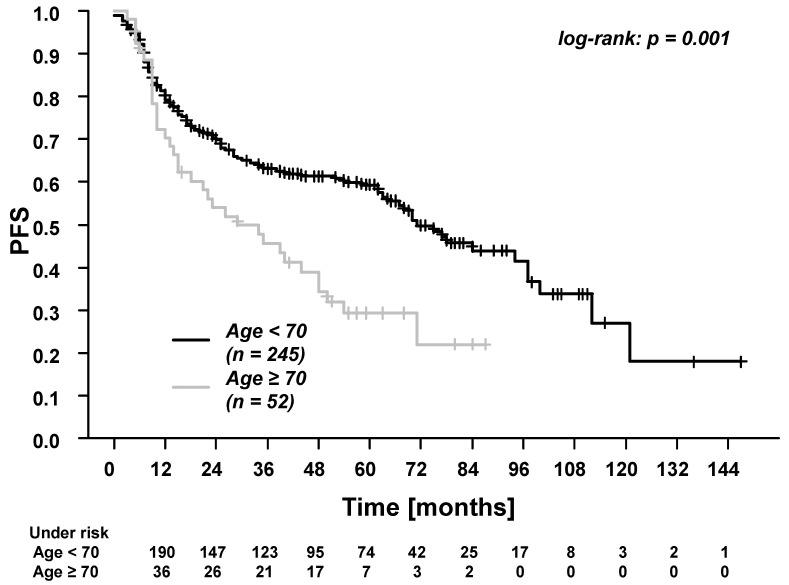
Progression-free survival (PFS) for patients <70 years vs. patients ≥70 years of age. The 3-year and 5-year PFS for patients <70 years were 63.0% and 59.2%. The 3-year and 5-year PFS for patients ≥70 years were 45.3% and 29.4%.

**Figure 3:**
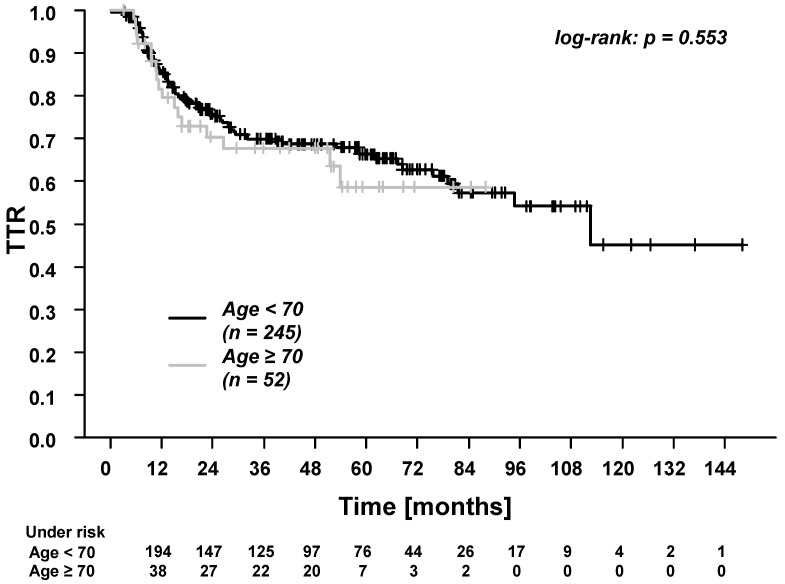
Time to recurrence (TTR) for patients <70 years vs. patients ≥70 years of age. The 3-year and 5-year TTR for patients <70 years were 69.9% and 66.3%. The 3-year and 5-year TTR for patients ≥70 years were 67.7% and 58.6%.

**Figure 4:**
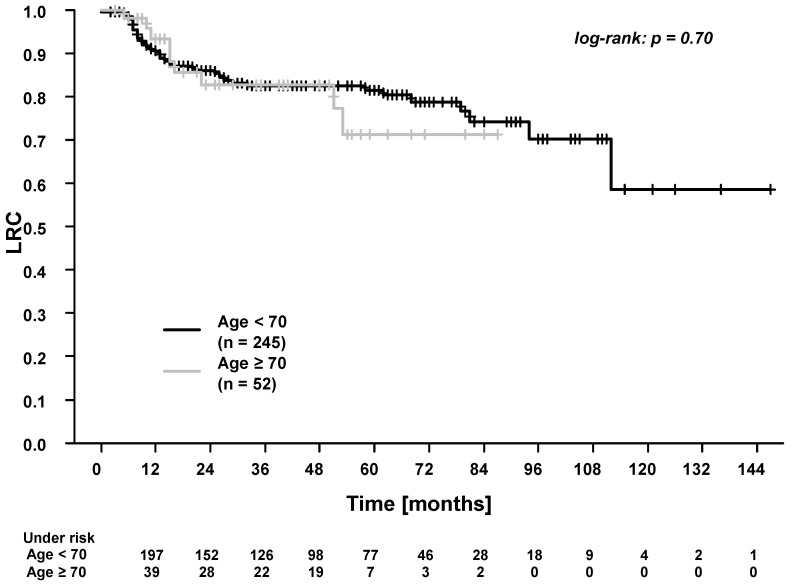
Locoregional control (LRC) for patients <70 years vs. patients ≥70 years of age. The 3-year and 5-year LRC for patients <70 years were 82.7% and 81.5%. The 3-year and 5-year LRC for patients ≥70 years were 82.7% and 71.2%.

**Figure 5:**
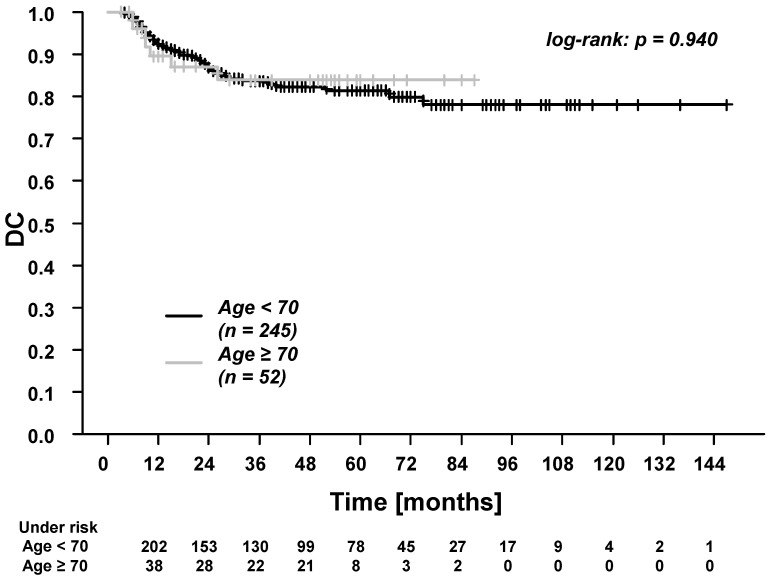
Distant control (DC) for patients <70 years vs. patients ≥70 years of age. The 3-year and 5-year DC for patients <70 years were 83.6% and 81.4%. The 3-year and 5-year DC for patients ≥70 years were both 83.9%.

In the whole cohort, the five-year overall survival (OS), progression-free survival (PFS), time to recurrence (TTR), locoregional control (LRC), and distant control (DC) were: 61.7%, 53.4%, 65.1%, 80.2%, and 81.6%. Patients ≥70 years experienced significantly worse OS and PFS (Figure 1 and Figure 2). There were no differences in TTR, LRC and DC (Figure 3, Figure 4 and Figure 5). During follow-up, locoregional recurrence occurred in 39/297 patients (14.1%). The locoregional recurrences were isolated primary tumor recurrences (*n* = 27 patients) and recurrences in the head and neck region outside the primary tumor site (*n* = 12 patients). Distant metastases were registered in 42/297 patients (14.1%). The metastases were localized in liver (*n* = 3 patients), lung (*n* = 24 patients), bone (*n* = 9 patients), and cerebrum (*n* = 6 patients). Additionally, in patients ≥70 years of age, there were no differences between patients who received radiotherapy alone vs. patients who received RCT (OS, TTR and LRC; Supplementary Figures S1–S3).

## 4. Discussion

In elderly patients, HNSCC are rare [9]. Less than 20% of HNSCC patients get diagnosed at the age of >70 years [9]. At the same time, regarding the general increase in life expectancy, the proportion of elderly patients with HNSCC is expected to grow rapidly [1,10]. Recently, several mostly retrospective studies focused on outcomes in elderly HNSCC patients and found evidence that specific management strategies are absolutely needed [13,14,15]. Nevertheless, until now, a deeper understanding of the implications of the findings for clinical routine was lacking [15]. In the present study, we analyzed patients treated with postoperative RCT for HNSCC at our tertiary cancer center. We compared baseline characteristics, treatment-associated toxicities, and outcomes between elderly patients ≥70 years of age and patients <70 years of age.

Firstly, we found no difference in locoregional control (LRC, five-year overall rate of 80.2%) between patients <70 years and patients ≥70 years of age. Additionally, there were no differences in radiotherapy completion rates, with >95% of elderly patients receiving 100% of the prescribed radiotherapy dose. These findings are comparable with the results of Haehl et al. in a study on 246 elderly HNSCC patients who received definitive or postoperative RCT [14]. Here, the authors report a two-year LRC rate of 75.5%, with 86.6% of patients completing the radiotherapy as planned [14]. In the present study, we found that elderly patients had node-negative disease more often. This is in line with the results of the study by Tomo et al., who reported similar findings in HNSCC patients >60 years of age [35]. In our study, the higher rates of tumors without nodal involvement might partly explain the excellent LRC rates in elderly patients [36]. The biological basis could be the more aggressive behavior of tumors in younger patients [37]. De Oliveira et al. found a higher expression of vascular endothelial growth factor-c in young patients with tongue cancers and hypothesized that this may play a role in age-dependent tumor behavior [37]. In summary, the excellent LRC and radiotherapy completion rates indicate that radiotherapy can be safely and effectively delivered after tumor resection in elderly patients with HNSCC.

Moreover, we found no difference in distant control (DC, five-year overall rate of 81.6%). The rates of DC were high for both younger (five-year, 81.4%) and elderly patients (5-year, 83.9%). Ahn et al. found comparable five-year DC rates of more than 80% in patients ≥70 years of age receiving definitive RCT for HNSCC [38]. In our study, these excellent tumor-related outcomes (LRC and DC) were achieved despite a smaller number of elderly patients receiving concomitant systemic treatment compared to the younger cohort. The careful use of concomitant systemic treatment in postoperative RCT of HNSCC was previously reported by Giacalone et al. [39]. Moreover, in the present study, concomitant systemic treatment could not be completed as planned in more elderly patients (54.8%) than younger patients (37.5%) (albeit not reaching statistical significance with a *p*-value of 0.07.). However, the missing information on total chemotherapy doses represents an important limitation. It should be mentioned that the major clinical trials which defined the standards for the use of concomitant systemic treatment in postoperative RCT for patients with HNSCC do not adequately reflect the elderly patient population. Bernier et al. excluded patients over 70 years [3]. In the study by Cooper et al., only 5% of patients were ≥70 years of age [4]. At the same time, elderly patients are at increased risk of side effects associated with systemic treatment (e.g., due to the age-related decrease in renal function when using cisplatin [40,41]). In summary, our study underlines that systemic treatment is a reasonable option in elderly patients, as stated by previous authors [42]. However, the indication should be carefully considered on an individual basis [42]. When comparing elderly patients treated with radiotherapy alone vs. elderly patients treated with RCT, we found no differences in toxicities, OS, TTR and LRC, possibly due to the low number of patients. As previous authors stated, cisplatin dose and schedule should be adopted to the specific clinical condition [38]. For example, daily low dose cisplatin can be an option in patients with relative contraindications for cisplatin (e.g., advanced age, reduced organ function) [28,43,44]. In our study, this regimen was used in 50% of the elderly patients.

Next, we found that patients ≥70 years experienced significantly worse overall survival (OS) and progression-free survival (PFS) than younger patients. The survival rate in patients <70 years was more than twice as good as in elderly patients (five-year OS, 68.7% vs. 33.8%). The lower survival rate in elderly patients can be attributed to the generally reduced life expectancy and to the higher prevalence of comorbidities [14]. In the present study, 98.1% of the elderly patients presented with a Charlson Cormorbidity Index of 4–7, which is associated with a 10-year survival rate from 0.01% (Index = 7) to 53.4% (Index = 4) [45]. However, due to the retrospective design of the present study, the causes of death were not systematically recorded. This represents a relevant limitation of the study. Nevertheless, the poor survival outcomes in patients ≥70 years highlight the need for thorough discussion of multimodal treatment options in this patient group [46].

In the present study, elderly patients experienced RCT-associated mucositis and dermatitis less frequently than younger patients. The careful use of concomitant systemic treatment in these patients might explain the lower rates of acute toxicities [47]. However, Müller von der Grün et al. and Singh et al. found no differences in the rates of acute toxicities of RCT when comparing younger and older HNSCC patients [48,49]. The heterogeneous results might be attributed to the fact that the studies on elderly patients only included a limited number of patients [14,48,49]. Furthermore, the studies differ in patient selection and radiotherapy technique [14,48,49]. Haehl et al. reported that ≥grade 3 dermatitis affected 19/246 patients (7.8%) and that ≥grade 3 mucositis affected 46/246 patients (18.7%) after treatment with 3D-conformal radiotherapy or IMRT (here, the proportion of patients was not further specified with regard to technique) [14]. Müller von der Grün et al. reported ≥grade 3 dermatitis for 15% of the elderly patients and ≥grade 3 mucositis for 49% of the elderly patients (≥70 years) [48]. Here, IMRT was used in 120/158 patients (75.9%) and 3D-conformal radiotherapy was used in 38/158 patients (24.1%) [48]. In the present study, all patients were treated with either IMRT or VMAT. This might explain the lower rates of ≥grade 3 dermatitis (elderly, 1.9%) and ≥grade 3 mucositis (elderly, 9.6%) when compared to the aforementioned studies. This indicates that modern radiotherapy techniques are important in effectively treating elderly HNSCC patients with, at the same time, moderate rates of acute toxicities [8,50].

## 5. Conclusions

Locally advanced HNSCC are often managed with surgery followed by postoperative RCT. With the general increase in life expectancy, the proportion of elderly patients with HNSCC is expected to grow rapidly. Until now, a deeper understanding of specific management strategies for these patients in clinical routines was lacking. In the present study, we compared elderly patients (≥70 years) and younger patients treated with postoperative RCT for HNSCC. All patients were treated with modern radiotherapy techniques (IMRT/VMAT). Patients ≥70 years of age had more comorbidities. Additionally, elderly patients less frequently received concomitant systemic treatment. The rates of RCT-associated mucositis and dermatitis were lower in patients ≥70 years. Elderly patients had significantly worse overall survival and progression-free survival. Locoregional and distant control were comparable in elderly and younger patients. In conclusion, postoperative RCT is a safe and effective treatment option in patients ≥70 years. In light of comorbidities and poor overall survival rates, benefits and harms of radiotherapy and concomitant systemic treatment should be weighed carefully. When exclusively applying up-to-date radiotherapy techniques with, at the same time, careful use of concomitant systemic therapy, favorable acute toxicity profiles are achieved.

## Figures and Tables

**Table 1:** Patient and tumor characteristics. For each parameter, either the number and percentage or the median and range are given. For comparison of treatment groups, we used the chi-square test.

Characteristics	Age, <70 Years (*n* = 245)	Age, ≥70 Years (*n* = 52)	*p*-Value
Age (years)	58 (23–69)	73 (70–85)	-
Sex			0.52
Female	47 (19.2)	8 (15.4)	
Male	198 (80.8)	44 (84.6)	
Charlson Comorbidity Index			<0.01
0–3	125 (51.0)	0 (0.0)	
4–7	119 (48.6)	51 (98.1)	
8–10	1 (0.4)	1 (1.9)	
Tumor localization			0.26
Hypopharynx	47 (19.2)	6 (11.5)	
Larynx	37 (15.1)	13 (25.0)	
Oral cavity	85 (34.7)	18 (34.6)	
Oropharynx	76 (31.0)	15 (28.8)	
HPV status			0.63
Positive	21 (27.6)	3 (20.0)	
Negative	29 (38.2)	6 (40.0)	
Undetermined	26 (34.2)	6 (40.0)	
Grading			0.76
G1	9 (3.7)	1 (1.9)	
G2	193 (78.8)	41 (78.8)	
G3	41 (16.7)	10 (19.2)	
Undetermined	2 (0.8)	0 (0.0)	
pT status			0.09
T0	5 (2.0)	4 (7.7)	
T1	38 (15.5)	6 (11.5)	
T2	61 (24.9)	12 (23.1)	
T3	85 (34.7)	13 (25.0)	
T4	56 (22.9)	17 (32.7)	
pN status			<0.01
N0	56 (22.9)	24 (46.1)	
N1	55 (22.4)	8 (15.4)	
N2	119 (48.6)	8 (15.4)	
N3	15 (6.1)	12 (23.1)	
Presence of ECE			0.36
Yes	64 (34.0)	12 (42.9)	
AJCC classification (8th edition, 2017) ^1^			0.86
I ^1^	18 (7.3)	3 (5.8)	
II ^1^	16 (6.5)	5 (9.6)	
III	75 (30.6)	15 (28.8)	
IV	136 (55.5)	29 (55.8)	
Resection status			0.59
R0	235 (95.9)	49 (94.2)	
R1	10 (4.1)	3 (5.9)	

^1^ In this study, the medical records were retrospectively reviewed and the AJCC stages were updated and harmonized according to the current 8th edition, including the classifications for HPV negative and HPV positive tumors. Consequently, 20 patients with HPV positive tumors were downstaged from the former classification which was applied for the treatment indications. Another 20 patients with stage I–II disease underwent surgery for recurrent tumors and subsequently underwent postoperative treatment. In 2 patients with stage I–II disease, the postoperative treatment was undertaken based on a discrepancy between initial clinical staging (cT3) and pathological staging (pT2) after transoral laser microsurgery.

**Table 2:** Treatment characteristics. For each parameter, either the number and percentage or the median and range are given. For comparison of treatment groups, we used the chi-square test* or the Mann–Whitney U test#.

Characteristics	Age, <70 Years (*n* = 245)	Age, ≥70 Years (*n* = 52)	*p*-Value
Radiotherapy			
Technique			0.77 *
VMAT	142 (58.0)	29 (55.8)	
IMRT	103 (42.0)	23 (44.2)	
Dose, planned [Gy]	64.0 (54.0–66.0)	64.0 (62.4–66.0)	0.47 #
Dose, administered [Gy]	64.0 (38.0–66.0)	64.0 (54.0–64.0)	0.33 #
Dose received			0.28 *
100% of planned dose	231 (94.3)	51 (98.1)	
≥80–<100% of planned dose	11 (4.5)	1 (1.9)	
<80% of planned dose	3 (1.2)	0 (0.0)	
Interruptions/breaks	117 (47.8)	29 (55.8)	0.29 *
Systemic treatment			
Received concomitant systemic treatment	192 (78.4)	31 (59.6)	0.04 *
Received <100% of planned dose	72 (37.5)	17 (54.8)	0.07 *
Systemic treatment type			<0.01 *
Cetuximab	3 (1.2)	3 (9.7)	
Cisplatin	189 (98.8)	28 (90.3)	
Cisplatin regimen			0.51 *
6 mg/m^2^/d daily	116 (61.4)	14 (50.0)	
20 mg/m^2^/d1–5 (2 cycles)	0 (0.0)	1 (3.6)	
40 mg/m^2^/d weekly	73 (38.6)	13 (46.4)	

**Table 3:** Toxicities. The CTCAE criteria and the LENT/SOMA criteria were used for the evaluation of acute and late toxicities. For each parameter, the number and percentage are given. For comparison of treatment groups, we used the chi-square test. * The information on late toxicity is missing in 30 patients.

Toxicities	Age, <70 Year (*n* = 245)	Age, ≥70 Years (*n* = 52)	*p*-Value
Acute organ toxicity			
Mucositis			0.03
0	13 (5.3)	9 (17.3)	
1	57 (23.3)	14 (26.9)	
2	148 (60.4)	24 (46.2)	
3	25 (10.2)	5 (9.6)	
4	2 (0.8)	0 (0.0)	
≥grade 3	27 (11.0)	5 (9.6)	0.91
Dermatitis			<0.01
0	6 (2.4)	7 (13.5)	
1	133 (54.3)	29 (55.8)	
2	100 (40.8)	15 (28.8)	
3	6 (2.4)	1 (1.9)	
≥grade 3	6 (2.4)	1 (1.9)	0.82
Dysphagia			0.23
0	29 (11.8)	4 (7.7)	
1	41 (16.7)	5 (9.6)	
2	48 (19.6)	14 (26.9)	
3	118 (48.2)	29 (55.8)	
4	9 (3.7)	0 (0.0)	
≥grade 3	127 (52.2)	29 (55.8)	0.64
Received feeding tube before RCT	64 (26.1)	19 (36.5)	0.13
Received feeding tube during RCT	65 (26.5)	15 (28.8)	0.73
Overall acute organ toxicity, ≥grade 3	137 (55.9)	30 (57.7)	0.82
Hematologic toxicity			
Overall hematologic toxicity, ≥grade 3	62 (25.3)	11 (21.2)	0.53
Anemia, ≥grade 3	6 (2.4)	2 (3.8)	0.57
Leukopenia, ≥grade 3	55 (22.4)	10 (19.2)	0.61
Thrombopenia, ≥grade 3	10 (4.1)	0 (0.0)	0.59
Overall late toxicity, ≥grade 2 *	90 (40.5)	17 (37.8)	0.73
Overall late toxicity, ≥grade 3 *	31 (14.0)	3 (6.7)	0.18

## Data Availability

The datasets generated during and/or analyzed during the current study are available from the corresponding author on reasonable request.

## Supplementary Materials

The following are available online at https://www.mdpi.com/article/10.3390/cancers13143384/s1, Table S1: Comparison of toxicities in patients ≥70 years of age, radiotherapy alone (RT) vs. radiochemotherapy (RCT). Figure S1: Comparison of overall survival (OS) in patients ≥70 years of age, radiotherapy alone (RT) vs. radiochemotherapy (RCT). Figure S2: Comparison of time to recurrence (TTR) in patients ≥70 years of age, radiotherapy alone (RT) vs. radiochemotherapy (RCT). Figure S3: Comparison of locoregional control (LRC) in patients ≥70 years of age, radiotherapy alone (RT) vs. radiochemotherapy (RCT).
