# Supplementary material for: Postoperative Radiochemotherapy Using Modern Radiotherapy Techniques in Elderly Patients with Head and Neck Squamous Cell Carcinoma: The Challenge of Weighing Up Benefits and Harms of Treatment Modalities in Clinical Practice
Source: Cancers (Basel). 2021 Jul 6;13(14):3384. doi: 10.3390/cancers13143384 (PMC8307771; doi:10.3390/cancers13143384)
Supplement: Supplementary file 1 [file cancers-13-03384-s001.zip › cancers-1214522-supplementary.pdf]

Supplementary Materials: Postoperative Radiochemotherapy Using Modern Radiotherapy Techniques in Elderly Patients with Head and Neck Squamous Cell Carcinoma: The Challenge of Weighing up Benefits and Harms of Treatment Modalities in Clinical Practice

Martin Leu, Christoph Patzer, Manuel Guhlich, Jacqueline Possiel, Yiannis Pilavakis, Markus Anton Schirmer, Stefan Rieken and Leif Hendrik Dröge

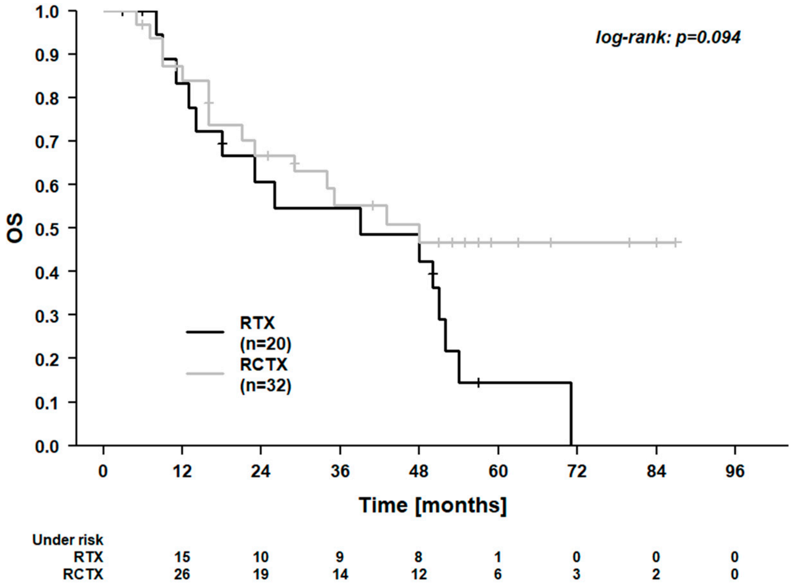

Figure S1. Comparison of overall survival (OS) in patients ≥70 years of age, radiotherapy alone (RT) vs. radiochemotherapy (RCT).

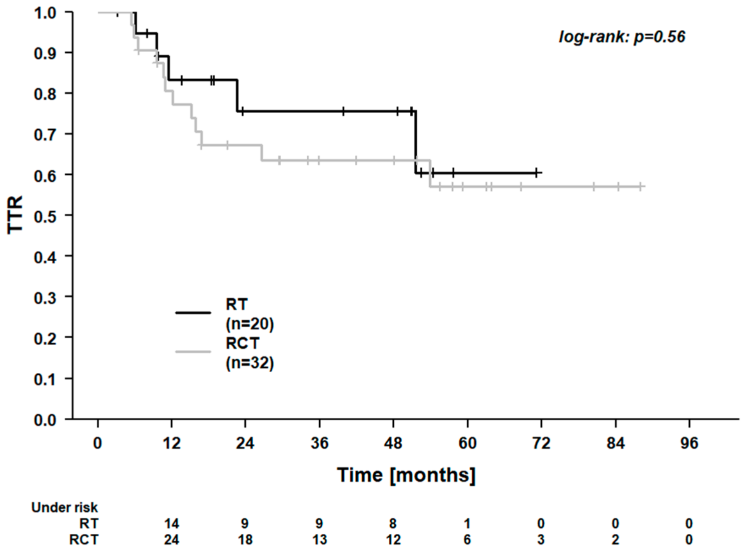

Figure S2. Comparison of time to recurrence (TTR) in patients ≥70 years of age, radiotherapy alone (RT) vs. radiochemotherapy (RCT).

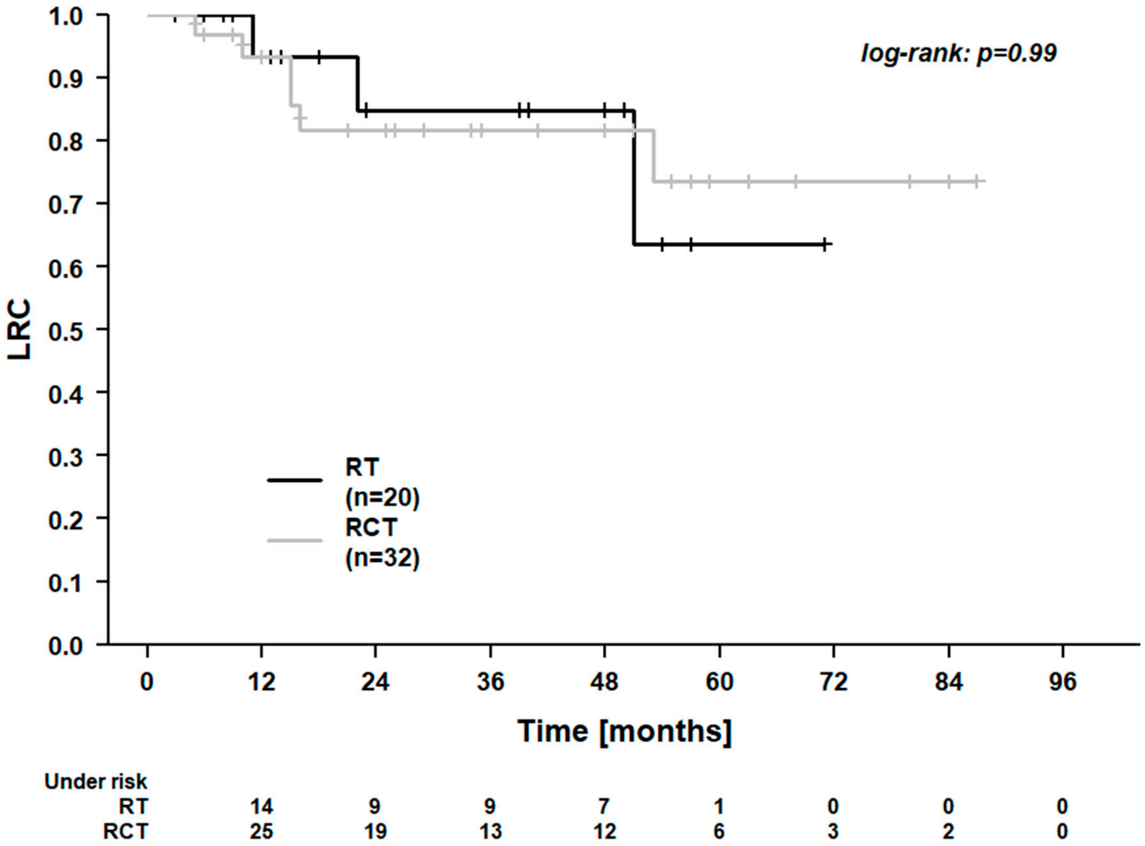

Figure S3. Comparison of locoregional control (LRC) in patients ≥70 years of age, radiotherapy alone (RT) vs. radiochemotherapy (RCT).

Table S1. Comparison of toxicities in patients ≥70 years of age, radiotherapy alone (RT) vs. radiochemotherapy (RCT). For each parameter, the number and percentage are given. For comparison of treatment groups, we used the chi-square test. * The information on late toxicity is missing in 14 patients.

	RT (*n* = 21)	(RCT *n* = 31)	p-Value
Mucositis			0.20
0	5 (23.8)	4 (12.9)	
1	8 (38.1)	6 (19.4)	
2	7 (33.3)	17 (54.8)	
3	1 (4.8)	4 (12.9)	
≥grade 3	1 (4.8)	4 (12.9)	0.71
Dermatitis			0.79
0	3 (14.3)	4 (12.9)	
1	11 (52.4)	18 (58.1)	
2	7 (33.3)	8 (25.8)	
3	0	1 (3.2)	
≥grade 3	0	1 (3.2)	0.41
Dysphagia			0.28
0	3 (14.3)	1 (3.2)	
1	1 (4.8)	4 (12.9)	
2	7 (33.3)	7 (22.6)	
3	10 (47.6)	19 (61.3)	
≥grade 3	10 (47.6)	19 (61.3)	0.33
Received feeding tube before RCT or during RCT	14 (66.7)	20 (64.5)	0.87

Received feeding tube before RCT	9 (64.3)	10 (50.0)	0.41
Overall acute organ toxicity, ≥grade 3	10 (47.6)	20 (64.5)	0.23
Overall late toxicity, ≥grade 2*	7 (41.2)	10 (35.7)	0.71
Overall late toxicity, ≥grade 3*	1 (4.8)	2 (6.5)	0.87
